# Diatomite silica nanoparticles for drug delivery

**DOI:** 10.1186/1556-276X-9-329

**Published:** 2014-07-03

**Authors:** Immacolata Ruggiero, Monica Terracciano, Nicola M Martucci, Luca De Stefano, Nunzia Migliaccio, Rosarita Tatè, Ivo Rendina, Paolo Arcari, Annalisa Lamberti, Ilaria Rea

**Affiliations:** 1 Department of Molecular Medicine and Medical Biotechnology, University of Naples Federico II, Naples 80131, Italy; 2 Institute for Microelectronics and Microsystems, National Council of Research, Naples 80131, Italy; 3 Department of Pharmacy, University of Naples Federico II, Naples 80131, Italy; 4 Institute of Genetics and Biophysics, National Council of Research, Naples 80131, Italy

**Keywords:** Diatomite, Porous silica nanoparticle, Drug delivery system, Surface modification

## Abstract

Diatomite is a natural fossil material of sedimentary origin, constituted by fragments of diatom siliceous skeletons. In this preliminary work, the properties of diatomite nanoparticles as potential system for the delivery of drugs in cancer cells were exploited. A purification procedure, based on thermal treatments in strong acid solutions, was used to remove inorganic and organic impurities from diatomite and to make them a safe material for medical applications. The micrometric diatomite powder was reduced in nanoparticles by mechanical crushing, sonication, and filtering. Morphological analysis performed by dynamic light scattering and transmission electron microscopy reveals a particles size included between 100 and 300 nm. Diatomite nanoparticles were functionalized by 3-aminopropyltriethoxysilane and labeled by tetramethylrhodamine isothiocyanate. Different concentrations of chemically modified nanoparticles were incubated with cancer cells and confocal microscopy was performed. Imaging analysis showed an efficient cellular uptake and homogeneous distribution of nanoparticles in cytoplasm and nucleus, thus suggesting their potentiality as nanocarriers for drug delivery.

**PACS:**

87.85.J81.05.Rm; 61.46. + w

## Background

Modern medicine has been revolutionized by the use of micro/nanocarriers that, acting theoretically as ‘magic bullets’ [1], operate in site-specific delivery mechanism to spare normal cells and tissues. A kind of natural microcarriers developed for innovative drug delivery is represented by diatomite silica microparticles [2]. Diatomite is a fossil material of sedimentary origin formed by fragments of diatom skeletons, called frustules. Frustules of diatoms, single-cell photosynthetic algae largely diffused in aquatic environments, are mainly constituted by amorphous silica and are characterized by a specific surface area up to 200 m^2^/g [3]. In nature, there are different kinds of diatoms (about 110,000 species) varying in size (from 2 μm to 2 mm) and morphology [4]. The low cost, abundance, easy availability, excellent biocompatibility, non-toxicity, thermal stability, and chemical inertness make diatomite an intriguing material for several applications ranging from filtration to pharmaceutics [5-8]. Diatomite is composed by 70 to 90% of silica, clay, some metallic oxides, such as Al_2_O_3_ and Fe_2_O_3_, and other organic components [4]. Usually, diatomite mined from geological deposits must be purified before to be used; thermal pre-calcination and HCl washing are the treatments generally used to increase powder quality and to make the biomaterial inert as filter support [9,10]. The diatomite silica surface presents reactive Si-OH groups that can be chemically modified in order to achieve a functionalized surface with proper chemical groups, such as − NH_2_, −COOH, −SH, and − CHO, which can be used for small interfering RNA (siRNA), microRNA (miRNA), decoy oligo, and drug loading [11,12].

In the present work, diatomite nanoparticles (DNPs) with a diameter lower than 300 nm were prepared by mechanical crushing, sonication, and filtering of micrometric diatomite powder. Nanoparticles, once purified from organic and inorganic impurities, were functionalized by using 3-aminopropyltriethoxysilane (APTES) and labeled with tetramethylrhodamine isothiocyanate (TRITC) in order to verify their cellular uptake. Confocal microscopy was used to investigate nanocarrier internalization in lung epidermoid cancer cells (H1355). Results demonstrated effective cellular uptake of nanoparticles and highlighted their potentiality in nanomedicine as carriers able to improve drug delivery.

## Methods

### Materials

Calcined diatomite was obtained by DEREF S.p.A (Castiglione in Teverina, Viterbo, Italy). 3-aminopro-pyltriethoxysilane (APTES), H_2_SO_4_, and tetramethylrhodamine isothiocyanate (TRITC) were purchased from Sigma-Aldrich (St. Louis, MO, USA). Phosphate-buffered saline (PBS) was purchased from GIBCO (Carlsbad, CA, USA). HCl was purchased from Romil (Cambridge, UK). Absolute ethanol and H_2_O_2_ was purchased from Carlo Erba (Milan, Italy); HEPES powder was purchased from Promega (Madison, WI, USA).

### Purification of diatomite powder

Five grams of crashed diatomite rocks were resuspended into 250 ml of absolute ethanol and sonicated for 5 h to break large aggregates. The dispersion was sieved through a nylon net filter with pore size of 41 μm, and then filtered with pore size of 0.45 μm (Millipore, Billerica, MA, USA). The diatomite nanopowder was purified to remove organic and inorganic impurities [9,10]. The sample was centrifuged and the pellet treated with Piranha solution (2 M H_2_SO_4_, 10% H_2_O_2_) for 30 min at 80°C. Nanoparticle dispersion was centrifuged for 30 min at 21,500 × *g*, washed twice with distilled water, resuspended in 5 M HCl, and incubated over night at 80°C. DNPs were then centrifuged for 30 min at 21,500 × *g* and washed twice with distilled water to eliminate HCl residues.

### Characterization of nanoparticles size

The size and zeta-potential measurements of purified diatomite nanoparticles dispersed in water (pH = 7) were performed before and after APTES functionalization by dynamic light scattering (DLS) using a Zetasizer Nano ZS (Malvern Instruments, Malvern, UK) equipped with a He-Ne laser (633 nm, fixed scattering angle of 173°, 25°C).

Transmission electron microscopy (TEM) and scanning electron microscopy (SEM) were also used to investigate nanoparticles morphology. Briefly, in TEM analysis, purified diatomite nanoshells were characterized by placing a drop of suspension on a TEM copper grid with a lacy carbon film and then observed by a Jeol 1011 TEM (Peabody, MA, USA) at an accelerating voltage of 100 KV. For SEM characterization, diatomite samples were deposited on crystalline silicon substrates mounted on a double-faced conductive adhesive tape. Images were acquired at 5-kV accelerating voltage and 30-μm wide aperture.

### Cell culture

The human lung epidermoid cancer cell line (H1355), obtained from American Type Tissue Collection (Rockville, MD, USA), was grown at 37°C with an atmosphere of 5% CO_2_, in RPMI 1640 (GIBCO) medium supplemented with 10% heat inactivated FBS (GIBCO), 100 U/mL penicillin, 100 mg/mL streptomycin, 1% l-glutamine.

### Diatomite functionalization

Purified nanoparticles were amino-modified with a 5% (*v*/*v*) APTES solution in absolute ethanol [13,14]. The APTES film formation was carried out for 1 h at room temperature with stirring in a dark condition. After this step, the sample was centrifuged for 30 min at 21,500 × *g* and supernatant discarded. The functionalized diatomite were washed twice with absolute ethanol and the collected pellet was incubated for 10 min at 100°C (curing process). Finally, the sample was washed twice with absolute ethanol and twice with 20 mM HEPES buffer pH 7.5.

### Fourier-transform infrared spectroscopy

Chemical composition of APTES-functionalized diatomite nanoparticles was analyzed by Fourier-transform infrared (FTIR) spectroscopy. Spectra were recorded by a Thermo-Nicholet NEXUS Continuum XL (Thermo Scientific, Waltham, MA, USA) equipped with a microscope, at 2 cm^−1^ resolution on samples deposited on silicon chips (*p*-type, 0.003 ohm cm resistivity, <100 > oriented, 500-μm tick) of about 1 cm × 1 cm.

### Nanopowder diatomite labeling

Diatomite labeling procedure was based on the use of an aminoreactive molecule, tetramethylrhodamine isothiocyanate. TRITC powder was solved in dimethyl sulfoxide (DMSO) and incubated with diatomite nanopowder in the presence of NaHCO_3_ 0.1 M pH 8.7 with stirring for 1 h at room temperature in a dark condition. Subsequently, the sample was washed with distilled water to remove TRITC excess, until no fluorescence was revealed in the supernatant when analyzed by fluorescence microscopy. Labeled diatomite nanoparticles will be indicated as DNPs*.

### Confocal microscopy

H1355 cell line (20 × 10^3^ cells/coverslip) was plated on 10-mm glass coverslips placed on the bottom of 24-well plate, allowed to attach for 24 h under normal cell culture conditions, and then incubated with increasing DNPs* concentration (5, 10, 15 μg/mL) for 24 h. As negative control, the last supernatant obtained from nanoparticles labeling procedure was added to the cells. Cell nuclei and membranes were then stained with Hoechst 33342 (Invitrogen, Carlslab, CA, USA) and WGA-Alexa Fluor 488, respectively. Images were acquired at × 63 magnification on a LSM710 confocal fluorescence microscope (Carl Zeiss Inc., Peabody, MA, USA) with the appropriate filters. Cell fluorescence intensity was analyzed by using ImageJ software (http://imagej.nih.gov/ij/).

## Results and discussion

### Characterization of diatomite nanoparticles

**Figure 1:**
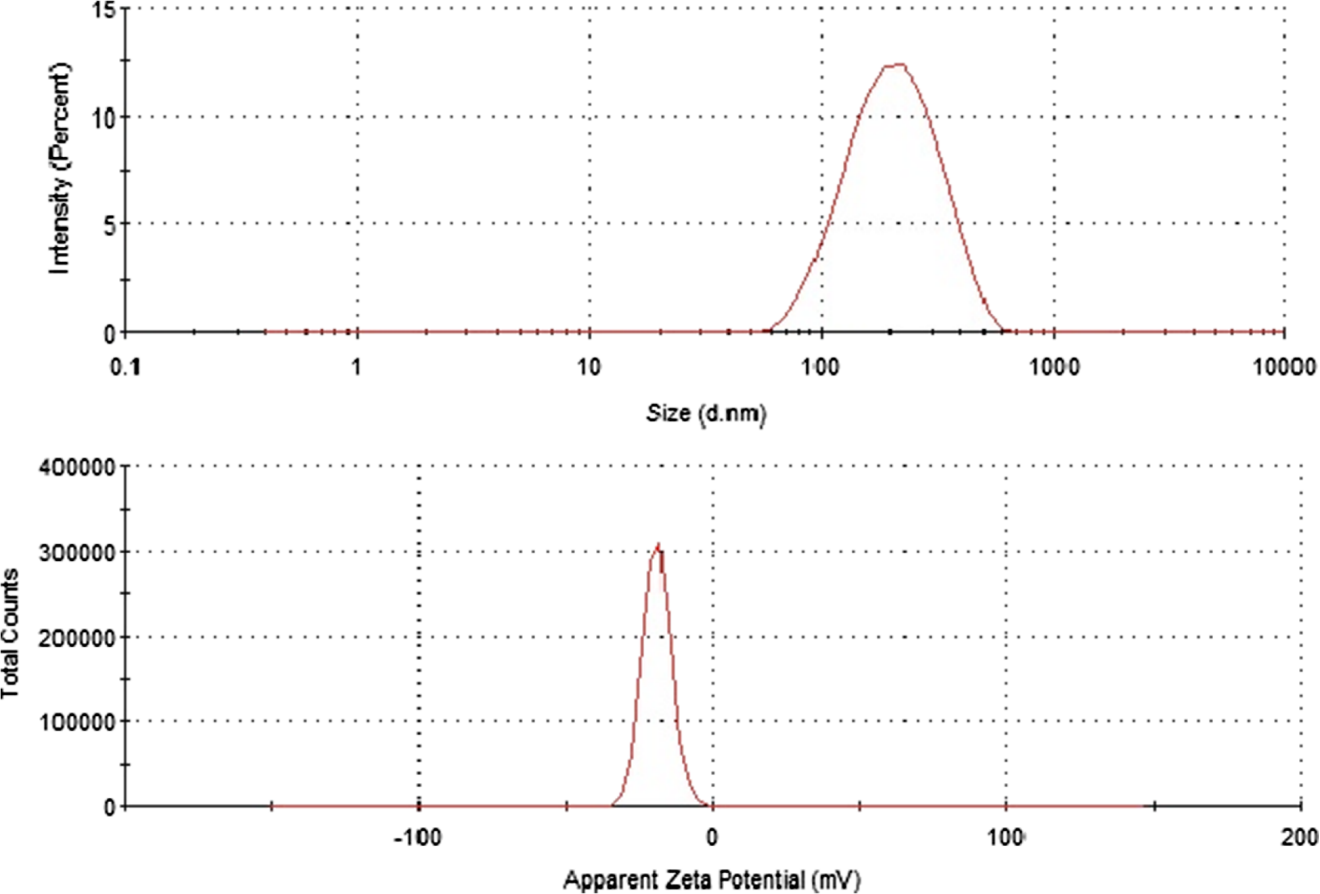
Size (upper graph) and zeta potential (lower graph) distributions of diatomite nanoparticles in water (pH = 7).

Size and surface charge of purified diatomite nanoparticles dispersed in water (pH = 7) were determined by DLS. The average size and zeta-potential of nanoparticles were 220 ± 90 nm and −19 ± 5 mV, respectively (Figure 1). The negative value of zeta-potential is due to the presence of silanol groups on nanoparticles surface after treatment in Piranha solution.

**Figure 2:**
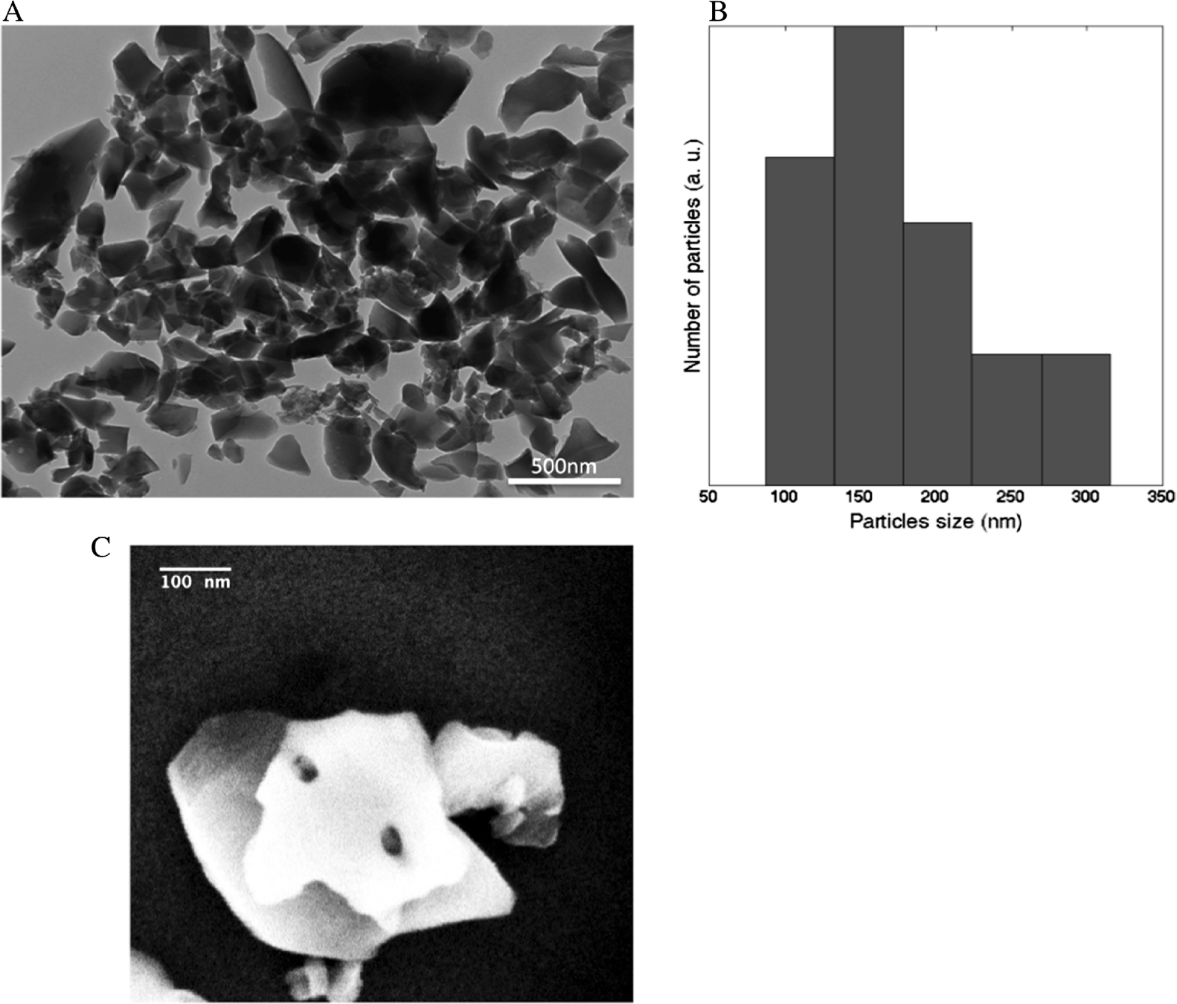
**TEM image, particles size distribution and SEM image of purified diatomite nanoshells.** Transmission electron microscopy image of DNPs **(A)** and particles size distribution **(B)** calculated from **(A)**. Scanning electron microscopy image of nanoparticle pores **(C)**.

Figure 2A shows a TEM image of purified diatomite nanoshells. A heterogeneous population constituted by nanostructures morphologically different in size and shape can be observed. The histogram of particle size, reported in Figure 2B and calculated from the picture reported in Figure 2A (by using ImageJ software), revealed a powder dimension ranging from 100 nm up to 300 nm with a maximum frequency value at 150 nm. The result was in agreement with that obtained by DLS analysis. The pore size of diatomite nanoparticles was estimated from SEM image reported in Figure 2C: pores of about 30 nm can be observed. Compared to other nanocarriers, the nanometric size and morphology of these particles make them suitable in drug delivery applications [15].

### Diatomite powder functionalization

**Figure 3:**
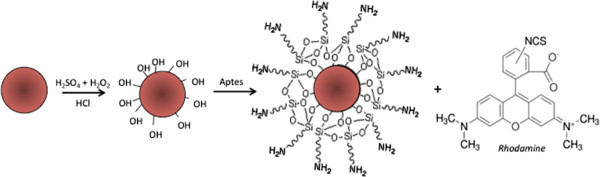
**Functionalization scheme of diatomite nanoparticles with rhodamine (TRITC).** APTES treatment allows surfaces substitution of the hydroxyl groups with − NH_2_ reactive amino-groups. These chemical modifications allow binding between − NH_2_ and rhodamine isothiocyanate group.

**Figure 4:**
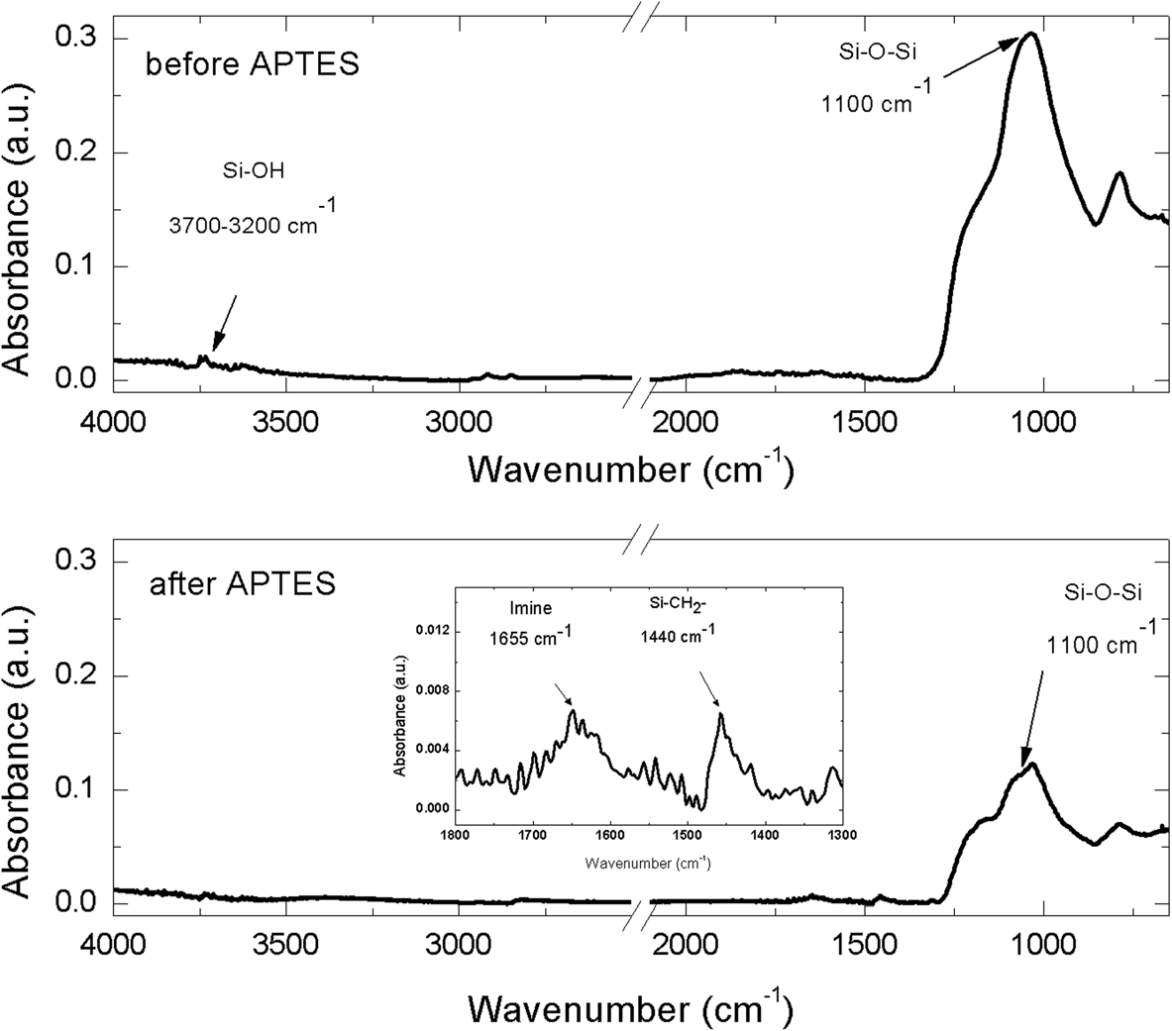
FTIR spectra of nanoparticles before (upper graph) and after (lower graph) APTES functionalization.

Hot acid-treated nanoparticles were functionalized with APTES solution to allow an amino-silane coating on their surface. The functionalization procedure is fully sketched in Figure 3. Silanol groups on diatomite surface were formed by hydroxylation using aqueous sulfuric acid. APTES in organic anhydrous solvent reacted with silanol groups on the activated surface producing siloxane linkages. Diatomite silanization was evaluated by FTIR spectroscopy. The comparison between FTIR spectra of bare nanoparticles (upper graph) and APTES-functionalized powders (lower graph) is reported in Figure 4. The peak of Si-O-Si bond at 1,100 cm^−1^, characteristic of diatomite frustules, is well evident in both spectra. Before APTES functionalization, it is also detected the peak at 3,700 to 3,200 cm^−1^ corresponding to Si-OH group. The spectrum of functionalized sample showed the silane characteristic peaks in the range between 1,800 and 1,300 cm^−1^ (see the inset of Figure 4); in particular, the peak at 1,655, corresponding to imine group and the peak at 1,440 cm^−1^, corresponding to asymmetric deformation mode of the CH_3_ group, were observed, according to results already reported [16,17]. FTIR characterization clearly demonstrated the silanization of silica nanoparticles.

APTES-modified silica nanoparticles dispersed in water (pH = 7) were also characterized by DLS analysis. A size of 280 ± 50 nm and a zeta-potential of +80 ± 5 mV were determined (data not shown). The positive potential is the result of protonation of amino groups on nanoparticles surface [18].

### Confocal microscopy analysis and DNPs* internalization

**Figure 5:**
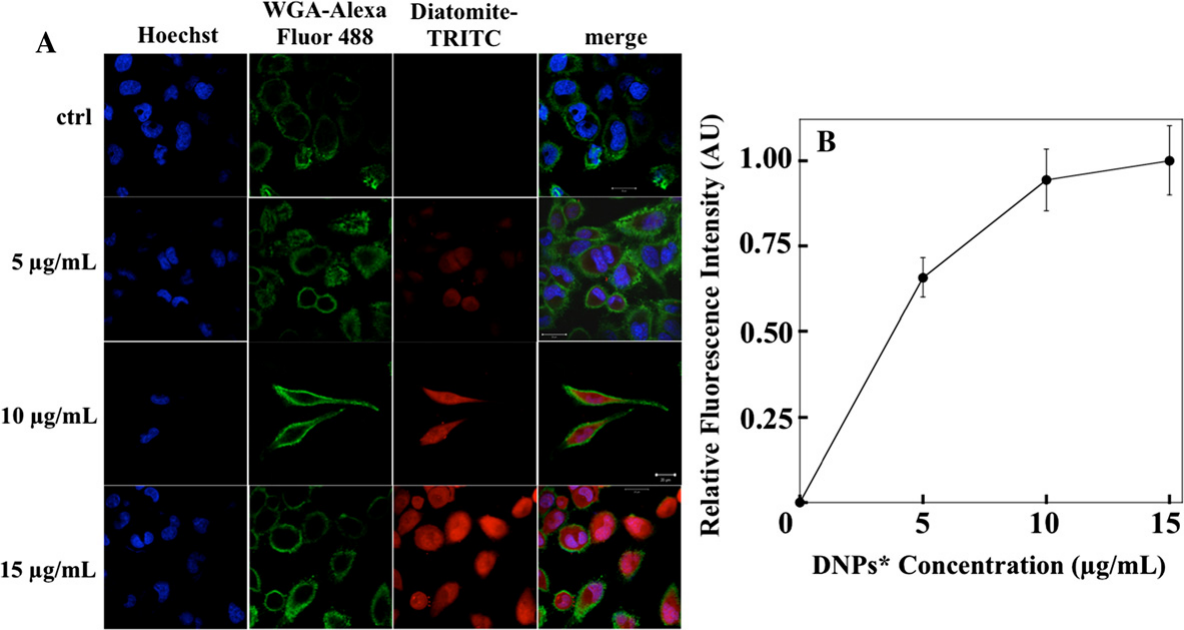
**Confocal microscopy images and cell fluorescence intensity analysis.** Confocal microscopy image of H1355 cells incubated with different concentrations of DNPs* **(A)**; scale bar corresponds to 20 μm. Cell fluorescence intensity *vs* nanoparticles concentration **(B)**; the values reported were obtained from fluorescence analysis of diatomite-TRITC images in panel **(A)**.

**Figure 6:**
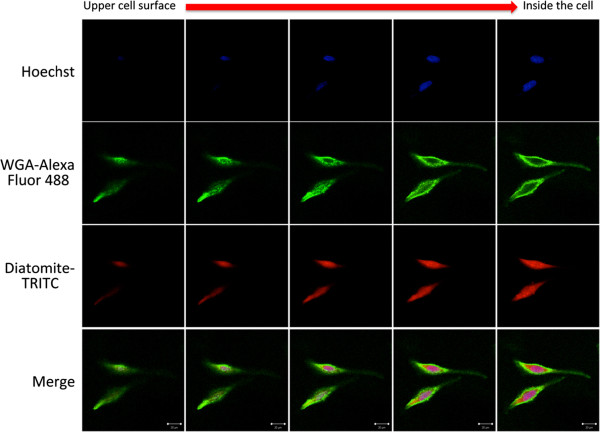
Confocal microscopy image with different focal depth of H1355 cells incubated with 10 μg/mL of DNPs*.

Nanoparticle cell uptake was studied by using DNPs* and confocal microscopy analysis. H1355 cells have been incubated with DNPs* at increasing concentrations (5, 10, 15 μg/mL) for 24 h. Figure 5A shows representative confocal microscopy images of cells treated with DNPs* compared to untreated cells as control. Cell nuclei were stained with Hoechst 33342 (blue), cell membranes were stained with WGA-Alexa Fluor 488 (green), and DNPs were labeled with TRITC (red). Images show an increase of fluorescence intensity at increasing DNPs* concentration and a homogeneous particles distribution in the cytoplasm and into nuclei. The cell fluorescence intensity *vs* labeled nanoparticles concentration is reported in Figure 5B; fluorescence values were calculated for each cell from the TRITC images of Figure 5A. Data showed an increase of the fluorescence intensity up to about 10 μg/mL. A saturation of the signal can be observed for nanoparticle concentrations higher than 10 μg/mL. To prove the internalization of the carriers in the cells, images at different focal depth were recorded. Figure 6 shows that going from upper cell surface to the focus inside the cells, an increase of red diatomite fluorescence can be observed thus indicating the uptake of DNPs* by H1355 cells.

## Conclusions

In this work, a procedure for preparing diatomite nanoparticles with an average size of 200 nm was described. DNP morphology and surface chemical modifications were investigated by DLS, SEM and TEM, and FTIR analyses, respectively. Confocal microscopy experiments revealed an efficient nanoparticle uptake into cytoplasm of human epidermoid carcinoma cells. This preliminary study demonstrates that the diatomite nanoparticles could represent a promising tool for the delivery of anticancer molecules such as siRNA, miRNA, and drugs inside cancer cells. Since APTES functionalization of the nanoparticles showed the possibility to efficiently bind amino-reactive groups (TRITC), the development of chemical protocols for loading anticancer molecules represents a further step in order to finalize the use of diatomite in medical applications. Moreover, it would be expected that compared to other nanocarriers, their selective targeted functionalization will improve the delivery of anti-tumoral molecules to specific cell population.

## Competing interests

The authors declare that they have no competing interests.

## Authors’ contributions

IR^1^ performed the experiments. IR^1^, AL, and IR^2^ designed the research. IR^1^ and AL analyzed data and wrote the paper. IR^2^ and LDS corrected the paper. RT assisted with confocal microscopy and transmission electron microscopy. MT prepared and characterized by dynamic light scattering the nanoparticles. NM performed cell culture. NMM participated in the experimental setup development and data analysis. IR and PA have given final approval of the version to be published. All authors read and approved the final manuscript.
